# Treatment with a DPP-4 inhibitor at time of hospital admission for COVID-19 is not associated with improved clinical outcomes: data from the COVID-PREDICT cohort study in The Netherlands

**DOI:** 10.1007/s40200-021-00833-z

**Published:** 2021-06-26

**Authors:** Rick I. Meijer, Trynke Hoekstra, Niels C. Gritters van den Oever, Suat Simsek, Joop P. van den Bergh, Renée A. Douma, Auke C. Reidinga, Hazra S. Moeniralam, Tom Dormans, Amsterdam UMC COVID-19 biobank study group, Mark M. Smits

**Affiliations:** 1 Department of Internal Medicine, Amsterdam UMC, Location AMC, Amsterdam, The Netherlands; 2 Department of Health Sciences and Amsterdam Public Health Research Institute, Vrije Universiteit Amsterdam, Amsterdam, The Netherlands; 3 Department of Intensive Care Medicine, Treant Zorggroep Emmen, Emmen, The Netherlands; 4 Department of Internal Medicine, Northwest Clinics, Alkmaar, The Netherlands; 5 Department of Internal Medicine, Amsterdam UMC, Location VUmc, Amsterdam, The Netherlands; 6 Department of Internal Medicine, Viecuri, Venlo, The Netherlands; 7 Department of Internal Medicine, Flevohospital, Almere, The Netherlands; 8 Department of Intensive Care Medicine, Martini Hospital, Groningen, The Netherlands; 9 Department of Internal Medicine, St. Antonius Hospital, Nieuwegein, The Netherlands; 10 Department of Intensive Care Medicine, Zuyderland Hospital, Heerlen, The Netherlands; 11 Amsterdam UMC, Location AMC, Amsterdam, The Netherlands; 12 Diabetes Center, Department of Internal Medicine, Amsterdam University Medical Center, Location Vumc, De Boelelaan 1117, 1081 HV Amsterdam, Netherlands

**Keywords:** COVID-19, Type 2 diabetes, DPP-4 inhibitors, Mortality

## Abstract

**Purpose:**

Inhibition of dipeptidyl peptidase (DPP-)4 could reduce coronavirus disease 2019 (COVID-19) severity by reducing inflammation and enhancing tissue repair beyond glucose lowering. We aimed to assess this in a prospective cohort study.

**Methods:**

We studied in 565 patients with type 2 diabetes in the CovidPredict Clinical Course Cohort whether use of a DPP-4 inhibitor prior to hospital admission due to COVID-19 was associated with improved clinical outcomes. Using crude analyses and propensity score matching (on age, sex and BMI), 28 patients using a DPP-4 inhibitor were identified and compared to non-users.

**Results:**

No differences were found in the primary outcome mortality (matched-analysis = odds-ratio: 0,94 [95% confidence interval: 0,69 – 1,28], *p*-value: 0,689) or any of the secondary outcomes (ICU admission, invasive ventilation, thrombotic events or infectious complications). Additional analyses comparing users of DPP-4 inhibitors with subgroups of non-users (subgroup 1: users of metformin and sulphonylurea; subgroup 2: users of any insulin combination), allowing to correct for diabetes severity, did not yield different results.

**Conclusions:**

We conclude that outpatient use of a DPP-4 inhibitor does not affect the clinical outcomes of patients with type 2 diabetes who are hospitalized because of COVID-19 infection.

## Introduction

The recent outbreak of severe acute respiratory syndrome coronavirus 2 (SARS-CoV-2), and its associated clinical condition called coronavirus disease 2019 (COVID-19), has led to a global threat of public health and economic systems. Patients with type 2 diabetes mellitus (T2DM) are particularly at risk of severe COVID-19 disease and adverse outcomes [1], presumably due to the associated obesity and hyperglycaemia. Whether poor glycaemic control at hospital admission and during hospital stay worsens the outcomes of COVID-19 remains a matter of debate [2, 3], yet is clear that absolute hyperglycaemia at time of admission increases this risk. Fascinatingly, those with newly diagnosed diabetes, with hyperglycaemia at admission, have higher risk of adverse outcomes [4]. Therefore, attention should be paid to optimise glucose regulation using glucose-lowering agents. Several medications are available to improve glucose regulation. Of those, the oral dipeptidyl peptidase (DPP)-4 inhibitors are of high interest in patients with COVID-19 for several reasons.

First, given certain similarities with Middle-Eastern Respiratory Syndrome coronavirus, it has been suggested that infection of respiratory cells by SARS-CoV-2 may be facilitated by DPP-4 [5], and inhibition might reduce its virulence. Second, DPP-4 inhibitors (DPP-4I) suppress systemic inflammation [6], and diminish tissue damage in a mouse model of lipopolysaccharide (LPS)-induced Acute Respiratory Distress Syndrome (ARDS) [7]. Since it is increasingly recognised that an over-responsive inflammatory reaction might be responsible for many of the adverse effects in COVID-19 [8], the use of DPP-4I could be protective. Third, DPP-4I increase levels of stromal-cell derived factor (SDF)-1, allowing faster tissue recovery due to stem cell mobilization [9].

A recent prospective non-randomised study by Solerte *et al.,* showed that starting sitagliptin at time of admission in T2DM patients with COVID-19 reduces mortality (hazard ratio 0.44), compared to standard care [10]. In contrast, cohort studies including patients already using DPP-4I treatment before hospitalisation have shown no harm, nor overt benefit of DPP-4I treatment, but did not correct for diabetes severity, which may confound the results. We therefore assessed whether use of DPP-4I prior to hospitalisation would be associated with improved outcomes compared with propensity matched controls.

## Methods

We used data from the ongoing CovidPredict Clinical Course Cohort, which contains prospective data from over 3200 patients with COVID-19 (www.covidpredict.nl). Patient data were (and are) obtained in 8 hospitals in the Netherlands. Patients with a positive SARS-CoV-2 PCR, or CT-scan abnormalities typical for COVID-19 (CO-RADS 4 and 5) [11], without another explanation for the abnormalities other than COVID-19, were included. The first patient was included on March 3^rd^ 2020, and for the current analysis, the last patient was included on October 1^st^ 2020 (to allow a follow-up of at least 40 days). The study protocol was reviewed by the medical ethics committees of the Amsterdam University Medical Centers (Amsterdam UMC; 20.131). Given the exceptional circumstances related to the COVID-19 crisis and in accordance with national guidelines and European privacy law, the need for informed consent was waived and opt out procedure was communicated by press release.

In this analysis, patients with T2DM were identified based on their medical history or the use of antihyperglycaemic agents (when type of diabetes mellitus was not specified, patients with insulin monotherapy were only included when having a BMI ≥ 30 kg/m^2^). The use of any DPP-4I was derived from medication parameters. No further selection of patients was applied, and patients of all ages and sex were included. The primary outcome was in-hospital mortality. Secondary outcomes included several aspects representing a more serious clinical course, including admission to the Intensive Care Unit, need for mechanical ventilation or non-invasive ventilation, the occurrence of thrombotic events, and infectious complications (pneumonia and sepsis). Furthermore, clinical data from the visit at the Emergency Room prior to admission were included as tertiary outcome: COVID-19 Reporting and Data System (CO-RADS)-score, CT-severity score, lab values and presence of pulmonary embolism.

Statistics were performed using Stata 16.0 SE. T2DM patients with DPP-4I were compared to T2DM patients without DPP-4I using several statistical techniques. First, we used both crude and propensity score matched analyses, to circumvent the limitations of the individual techniques. Propensity score matching was performed using the ‘teffects psmatch’ command in the regression analyses [12]. Matching was performed for age, BMI and sex (thus allowing simultaneous correction for these factors, hereby approximating treatment effects between groups in non-randomized data). Both crude and matched analyses were performed with logistic regression (for binomial variables, including the primary and secondary outcomes) and linear regression (for continuous outcomes, mainly lab values as tertiary outcomes). The non-matched analyses were additionally performed after correction for known confounders of COVID-19 outcome: age, sex and comorbidity.

Importantly, since diabetes control and its complications may be related to COVID-19 outcomes, correction for diabetes severity was deemed necessary. As the database did not include data on HbA1c or diabetes related complications, we decided to compare the group of all DPP-4I users (with or without other antihyperglycaemic medication) with (1) patients only using metformin ± sulphonylurea, and with (2) patients using insulin, with or without antihyperglycaemic agent (other than DPP-4I). When following national and international guidelines, patients using DPP-4I will form an intermediate group with regards to diabetes control and complications compared to those two treatment groups. Comparing the different groups was performed for all analyses (i.e. propensity score matched, crude and corrected analyses).

## Results

In total, 565 patients with T2DM admitted with COVID-19 were identified in our cohort. Of these, 28 patients (5%) used a DPP-4I. Characteristics of the DPP-4I group, as well as the non-DPP-4I groups are shown in Table 1. As expected, and in accordance with Dutch guidelines, DPP-4I users were frequently cotreated with metformin and/or sulphonylurea agents. Compared with non-DPP-4I users, there was no clinically relevant or statistical difference in age, sex, BMI or blood pressure. Patients treated with insulin were more likely to have comorbidities.

**Table 1:** Characteristics of the studied groups, mean with standard deviation

	DPP4 +	Group 1: Non-DPP-4	*P-value*	Group 2: Metformin + SU	*P-value*	Group 3: Insulin ± combination	*P-value*
	*N* = *28*	*N* = *537*		*N* = *104*		*n* = *140*	
Demographics							
Age (yrs)	66,88 (12,41)	69,48 (12,5)	0,282	68,9 (11,4)	0,412	68,69 (12,4)	0,475
Sex (% male)	60,70%	64,2	0,704	67,3	0,514	61,1	0,971
Health							
BMI (kg/m^2^)	29,09 (6,0)	29,84 (6,3)	0,579	29,11 (5,1)	0,988	30,56 (7,4)	0,527
SBP (mmHg)	140,14 (26,2)	136,65 (25,3)	0,479	137,24 (32,0)	0,66	136,98 (24,9)	0,539
DBP (mmHg)	80,68 (20,83)	76,08 (17,1)	0,17	76,5 (22,6)	0,383	74,73 (16,6)	0,093
Comorbidity (%)							
Any	81,50	88,1	0,055	83,5	0,223	89	0,043*
Cardiac	48,10	41,1	0,465	39,8	0,434	42,8	0,601
Hypertension	66,70	70	0,716	72,1	0,578	71,9	0,581
Pulmonary	14,30	28,2	0,108	25,7	0,204	28,7	0,109
Kidney	25,90	14,4	0,103	12,7	0,092	19,3	0,425
Liver	0,00	1,9	0,471	2	0,461	1,8	0,483
Neurological	7,10	16	0,206	15	0,279	12	0,449
Neoplasm	0,00	7,2	0,15	5,9	0,197	10,2	0,083
Haematologic	7,40	3	0,21	2	0,153	5,4	0,68
HIV/AIDS	0,00	1,1	0,579	3	0,365	1,2	0,568
Rheumatologic	7,40	15,8	0,238	7,9	0,93	19,8	0,121
Dementia	3,60	5,9	0,609	3	0,871	7,8	0,424
Medication (%)							
DDP4i	100	0	N/A	0	N/A	0	N/A
Metformine	67,90	77,1	0,26	100	N/A	76	0,355
Sulfonylurea	67,90	32,2	< 0,001*	100	N/A	28,1	< 0,001*
GLP-1RA	0,00	2	0,444	0	N/A	3,6	0,308
SGLT2i	0,00	1,3	0,543	0	N/A	0,6	0,681
Insulin	17,90	31,1	0,138	0	< 0,001*	100	N/A
Outcome (%)							
Death	29,60	32,8	0,734	32,6	0,768	39,9	0,313
ICU	32,10	22,7	0,249	29,8	0,811	21,6	0,219
Invasive ventilation	25,00	19,4	0,465	26	0,918	16,8	0,293
Noninvasive ventilation	3,57	8	0,393	12,5	0,173	7,2	0,478
Any thrombotic event	7,10	7,4	0,952	12,5	0,428	6	0,814
Pulmonary embolism	3,60	3,2	0,905	3,8	0,946	3,6	0,996
Bacterial pneumonia	7,10	7,8	0,896	7,7	0,992	9,6	0,68
Septic shock	3,60	7,8	0,408	8,7	0,367	7,2	0,478
Characteristics at admission							
Hemoglobin (mmol/L)	7,92 (1,2)	8,08 (3,8)	0,851	7,84 (1,2)	0,779	8,22 (6,6)	0,839
Leukocyte (10^9^/L)	8,82 (3,9)	8,05 (4,5)	0,425	7,71 (3,5)	0,191	7,90 (3,6)	0,266
Platelets (10^9^/L)	263,09 (86,6)	225,20 (90,8)	0,051	244,89 (89,8)	0,387	214,76 (97,0)	0,026*
ALAT (U/L)	41,45 (29,2)	47,99 (150,8)	0,847	40,22 (26,4)	0,856	42,57 (43,7)	0,912
ASAT (U/L)	60,06 (75,5)	63,16 (145,0)	0,993	52,59 (33)	0,553	59,54 (62,1)	0,976
Glucose (mmol/L)	13,58 (8,9)	10,85 (4,8)	0,022*	12,11 (5,8)	0,406	11,38 (4,8)	0,114
Bilirubin (umol/L)	7,94 (2,98)	10,61 (9,9)	0,257	9,57 (5,1)	0,2	11,91 (15,8)	0,291
Lactate (mmol/L)	2,06 (1,7)	2,1 (1,7)	0,906	1,83 (0,8)	0,546	2,14 (1,8)	0,893
Creatinine (umol/L)	132,91 (160,7)	123,51 (116,0)	0,71	121,51 (132,1)	0,726	129,3 (105,5)	0,89
CRP (mg/L)	69,79 (55,1)	110,4 (91,2)	0,042*	128,46 (90,7)	0,005*	104,24 (81,7)	0,017*
LDH (U/L)	320,56 (78,9)	379,43 (185,2)	0,207	379,26 (170,2)	0,184	362,5 (175,6)	0,35
D-dimer (mg/L)	1096,82 (2007,8)	1298,23 (4414,5)	0,912	304,99 (1008,8)	0,211	450,49 (603,1)	0,468
CT Severity Score	9	9,78 (5,9)	0,678	11 (6,1)	0,341	362,5 (175,6)	0,993
CT CO-RADS score (median)	5	5	0,848	5	0,458	5	0,956
Pulmonary Embolism (%)	3,6	0,6	0,064	1	0,316	0	0,014*

Legend: characteristics of the group of patients using a DPP-4I versus the different groups of patients not using a DPP-4I. Importantly, this table includes data of all included subjects, not limited to those included in the propensity-matched analysis. The given *p*-values therefore represent the unmatched and non-corrected ‘crude’ analyses between the given groups

* an asterisk indicates a statistical significant difference between the groups

After propensity score matching, the use of DPP-4I at time of admission was not associated with lower odds of in-hospital death compared to any of the non-DPP-4 groups (group 1, all non-DPP-4 users: OR = 0.93 and p = 0.689; group 2, patients using metformin and sulphonylurea but no DPP-4I: OR = 0.94, and p = 0.647; group 3, patients insulin with or without other antihyperglycaemic agents, but no DPP-4I: OR = 0.85 and p = 0.373; Table 2). The odds of secondary outcomes, i.e. the need for ICU admission, or invasive ventilation, occurrence of thrombotic events, and infectious complications (pneumonia and sepsis), were not different between DPP-4I and non-DPP-4I users (Table 2).

**Table 2:** Regression analyses

	Propensity score matched		Unmatched uncorrected		Unmatched corrected for age, sex, BMI and comorbidity	
	Odds Ratio + 95% CI	*p*-value	Odds Ratio + 95% CI	*p*-value	Odds Ratio + 95% CI	*p*-value
Mortality						
(1) DPP-4I vs all non-DPP-4I	0,93 (0,68 – 1,28)	0,689	0,86 (0,36 – 2,01)	0,734	1,00 (0,36 – 2.82)	0.990
(2) DPP-4I vs metformin/SU	0,94 (0,73 – 1,21)	0,647	0,86 (0,34 – 2,2)	0,768	0,86 (0,27 – 2,73)	0,797
(3) DPP-4I vs Insulin*	0,85 (0,59 – 1,21)	0,373	0,63 (0,26 – 1,54)	0,316	0,74 (0,25 – 2,18)	0,591
ICU admission						
(1) DPP-4I vs all non-DPP-4I	0,99 (0,8 – 1,22)	0,95	1,61 (0,71 – 3,65)	0,253	1,26 (0,48 – 3,30)	0,638
(2) DPP-4I vs metformin/SU	0,94 (0,74 – 1,2)	0,648	1,11 (0,45 – 2,73)	0,811	0,83 (0,29 – 2,35)	0,722
(3) DPP-4I vs Insulin*	1,04 (0,9 – 1,2)	0,538	1,72 (0,71 – 4,13)	0,222	1,66 (0,58 – 4,76)	0,347
Invasive ventilation						
(1) DPP-4I vs all non-DPP-4I	0,98 (0,81 – 1,19)	0,911	1,38 (0,57 – 3,35)	0,466	1,23 (0,46 – 3,32)	0,677
(2) DPP-4I vs metformin/SU	0,9 (0,71 – 1,14)	0,396	0,95 (0,36 – 2,48)	0,918	0,85 (0,28 – 2,51)	0,761
(3) DPP-4I vs Insulin	1,05 (0,9 – 1,22)	0,507	1,65 (0,64 – 4,26)	0,297	1,94 (0,65 – 5,79)	0,237
Thrombotic events						
(1) DPP-4I vs all non-DPP-4I	0,98 (0,9 – 1,07)	0,804	0,95 (0,21 – 4,17)	0,952	0,45 (0,06 – 3,45)	0,447
(2) DPP-4I vs metformin/SU	0,89 (0,75 – 1,05)	0,178	0,53 (0,11 – 2,53)	0,434	0,24 (0,03 – 1,95)	0,237
(3) DPP-4I vs Insulin*	0,96 (0,88 – 1,06)	0,515	1,2 (0,25 – 5,82)	0,814	0,58 (0,06 – 5,52)	0,622
Infectious events						
(1) DPP-4I vs all non-DPP-4I	0,93 (0,76 – 1,15)	0,536	0,74 (0,21 – 2,54)	0,638	0,73 (0,20 – 2,59)	0,624
(2) DPP-4I vs metformin/SU	0,94 (0,79 – 1,11)	0,485	0,67 (0,18 – 2,54)	0,565	0,64 (0,16 – 2,54)	0,528
(3) DPP-4I vs Insulin*	0,9 (0,73 – 1,1)	0,318	0,63 (0,17 – 2,28)	0,488	0,70 (0,18 – 2,71)	0,603

Legend: regression analyses on the primary outcome (mortality) and secondary outcomes (ICU admission, invasive ventilation, thrombotic events or infectious events). Thrombotic events include diffuse intravasal coagulation, deep venous thrombosis, pulmonary embolism and cerebral venous sinus thrombosis. Infectious events include the occurrence of pneumonia and septic shock. For all outcomes, both the propensity-score matched analyses and unmatched analyses are shown for the different groups

^*^in this group, insulin users were included, irrespective of the use of other anti-hyperglycaemic agents, except for DPP-4I

Because of the relatively limited numbers of patients, we also performed crude analysis without propensity score matching, which did not yield different results (Table 2). Moreover, after correction for age, sex, BMI and co-morbidity, there was no difference in mortality or secondary outcomes between the DPP-4I group and the groups that did not use DPP-4I (Table 2).

Interestingly, patients using DPP-4I had lower CRP and lower LDH levels at time of admission compared to the other groups (Table 1). Although there appeared to be a tendency towards more frequent diagnosis of pulmonary embolism at admission (a higher proportion of DPP-4I compared to the whole-group of non-users, and statistically significant compared with the non-DPP-4I insulin users), there was no difference in the incidence of this diagnosis at hospital discharge.

## Discussion

Based on the proposed interaction of SARS-CoV-2 with DPP-4 [5], and the effects of DPP-4I on inflammation and glycaemic control [6, 7, 9], we hypothesised that patients with T2DM using DPP-4I at time of hospital admission with COVID-19 would have improved outcomes. Our data however do not support this, as we have found no association with mortality nor a more severe clinical course.

While conducting our analysis, several other groups published their data focusing on the same hypothesis. In the last months, multiple meta-analyses were performed with contrasting results. In one study, incorporating 8 retrospective and prospective cohort studies and 1 case series, concluded that use of DPP-4 inhibitors was associated with 24% lower mortality in patients with COVID-19 (risk ratio 0.76, 95%-CI 0.60–0.97) [13]. Apart from the prospective cohort study performed by Solerte et al. [10], none of the included studies demonstrated a statistical significant effect on their own. A different recent meta-analysis on 10 studies, with some overlap with the before mentioned study, showed no effect on composite poor outcomes, or mortality (OR 1.14, 95%-CI 0.87–1.51) [14]. In one cohort study from Singapore, use of DPP-4I actually demonstrated an increased risk of ICU admission (odds ratio of 3.3) of versus non-users [15].

Importantly, what separates our study from other published data to date, is the method to correct for diabetes severity. In other studies, users of DPP-4I were compared to non-users, and frequently statistical correction was applied for age, sex, and comorbidities. Correction for diabetes severity was however not applied. Importantly, T2DM patients can be heterogenous with regards to diabetes severity, glucose control and complications. As such, patients only using metformin are generally not comparable to those on a multi-dose insulin schedule, while both groups do not use DPP-4I. Simply comparing DPP-4I users to non-users could introduce bias when not correcting for diabetes severity. Therefore, in the current analysis, we compared DPP-4I users to several groups of non-DPP-4I users, hereby creating a surrogate for diabetes severity. Using this technique, we observed no difference in outcomes between the groups.

The current analysis has several limitations. First, and foremost, the absolute number of patients using a DPP-4I is relatively low. However, we used propensity score matching to increase statistical power by generating comparable groups. Moreover, even though the numbers are low, it is one of the largest prospective analyses in a Western population. Second, the database did not contain data on diabetes duration, or the duration of taking DPP-4 inhibitors. As indicated, we have tried to circumvent the lack of information on diabetes duration, diabetes complications, or glucose control by using other diabetes medication as proxy. A strength of the current study is the use of prospective data from several hospitals throughout The Netherlands, without selection or inclusion criteria regarding diabetes, which allows generalization to most (Dutch) patients with T2DM.

Thus, it can be suggested that patients with T2DM who already use DPP-4I do not have a different clinical course when contracting COVID-19, yet more studies are needed to understand whether starting a DPP-4I at time of admission is beneficial.

## Data Availability

Upon request at the corresponding author.
